# The Butterfly Effect of RNA Alterations on Transcriptomic Equilibrium

**DOI:** 10.3390/cells8121634

**Published:** 2019-12-13

**Authors:** Ng Desi, Yvonne Tay

**Affiliations:** 1 Cancer Science Institute of Singapore, National University of Singapore, Singapore 117599, Singapore; cside@nus.edu.sg; 2 Department of Biochemistry, Yong Loo Lin School of Medicine, National University of Singapore, Singapore 117597, Singapore

**Keywords:** post-transcriptional regulation, RNA alteration, microRNA, competing endogenous RNA, RNA-binding protein, cancer, transcriptomic equilibrium

## Abstract

Post-transcriptional regulation plays a key role in modulating gene expression, and the perturbation of transcriptomic equilibrium has been shown to drive the development of multiple diseases including cancer. Recent studies have revealed the existence of multiple post-transcriptional processes that coordinatively regulate the expression and function of each RNA transcript. In this review, we summarize the latest research describing various mechanisms by which small alterations in RNA processing or function can potentially reshape the transcriptomic landscape, and the impact that this may have on cancer development.

## 1. Introduction

Our understanding of the molecular biology of gene regulation has come a long way since Francis Crick coined the term “the central dogma” in 1957 [1]. While much early research focused on DNA and proteins, an increasing amount of attention in recent years has been placed on RNA biology. The advent of next generation sequencing technologies has led to the discovery of multiple novel RNA species, and a paradigm shift from the classical view of RNA as an intermediary for protein synthesis to a deeper appreciation of the multi-faceted roles that RNAs play in key cellular processes and the pathogenesis of diseases including cancer. Moreover, the increased transcript and protein expression of key oncogenes, in the absence of genomic alterations, has been linked to cancer development in a subset of patients. This suggests that the dysregulation of RNA expression may play a crucial role in cancer development [2,3]. Indeed, there have been numerous reports describing the perturbation of post-transcriptional regulatory processes in cancer [4,5]. In addition to affecting RNA expression, some of these regulators can modify RNA length and nucleotide integrity, thereby influencing RNA function. Moreover, the disruption of key post-transcriptional processes in cancer may directly and/or indirectly affect multiple target RNAs to establish cancer initiation and enhance progression, thus creating the butterfly effect.

## 2. MicroRNAs: Small, But Powerful Post-Transcriptional Regulators

Since the discovery of microRNAs in C. elegans and subsequently in humans, there has been an explosion of interest in this large class of small non-coding RNAs [6,7]. This was amplified when microRNAs were first linked to human disease; that is, when Calin et al. [8] demonstrated that miR-15 and miR-16 were frequently deleted in leukemia. Multiple subsequent publications have corroborated the link between dysregulated miRNA expression and cancer progression. Some miRNAs such as the Let-7 family, miR-34 family, and miR-138 have been validated by several independent studies as tumor suppressive miRNAs, which are frequently downregulated in cancers compared with normal tissues [9,10,11]. On the other hand, the upregulation of oncogenic miRNAs (also known as oncomiRs), such as miR-21, the miR-17/92 cluster, and the miR-106b/25 cluster, has been shown to promote various hallmarks of cancer, leading to more aggressive disease, poorer prognosis, and lower survival in cancer patients [12,13,14].

Other than changes in specific miRNA levels, the dysregulated expression of miRNA biogenesis-associated nucleases Drosha and Dicer has been linked to cancer [15,16]. MicroRNA biogenesis involves a cascade of meticulously orchestrated processing events, starting from the transcription of primary miRNAs (pri-miRNAs) from the introns of host genes or as independent transcriptional units. Cleavage by the DROSHA microprocessor complex produces 55–70 nucleotide long precursor miRNA (pre-miRNA) hairpins [17,18]. The cleaved pre-miRNAs are exported by Exportin-5 to the cytoplasm, where they are trimmed by the RNase III enzyme DICER to yield mature miRNAs, which are then incorporated into the RNA-induced silencing complex (RISC) [19]. Additionally, it has been reported that some miRNAs are processed through a non-canonical DICER-independent pathway, which is mediated by Argonaute2 (AGO2) [20]. Although miRNAs were initially considered to be distinct from other classes of small non-coding RNAs, such as snRNAs, snoRNAs, and tRNAs, which have relatively well-characterized roles in RNA processing and translation, recent studies have shown that small RNAs derived in a DICER-dependent manner from tRNAs and snoRNAs can associate with AGO proteins and exhibit microRNA-like functionality [21].

MiRNA binding usually leads to transcript degradation or translational repression, depending on the degree of complementarity with target transcripts. This imperfect nature of miRNA/target base pairing has resulted in significant challenges in predicting miRNA targets in eukaryotes. Nucleotides 2–8 from the 5-end of a miRNA (5′ seed region) generally facilitate the recognition of miRNA response elements (MREs) on target RNAs, while nucleotides 10–11 have been shown to mediate the endonucleolytic cleavage by AGO2 [22,23,24,25,26]. Intriguingly, multiple studies have reported the existence of non-canonical miRNA/target interactions that are independent of 5′ seed binding, suggesting that miRNA species may systematically differ in how they interact with target transcripts [27,28]. Further studies showed that these non-canonical miRNA/target interactions are widespread and also resulted in target transcript repression, albeit generally weaker than canonical interactions [29]. Surprisingly, the extensive 3′ pairing between miRNAs and their non-canonical targets enhances the 3′ end destabilization of miRNAs within AGO proteins [30]. These findings underscore our limited understanding of miRNA target recognition and specificity, which is of fundamental importance in miRNA-mediated post-transcriptional regulation.

As the first few miRNA/target interactions were shown to involve binding at the 3′ untranslated regions (UTRs) of messenger RNAs (mRNAs), a significant proportion of subsequent research has focused on this class of miRNA targets [31]. However, miRNA targeting is not limited to 3′UTRs—they have been shown to regulate the coding regions and 5′UTRs of mRNAs, as well as multiple non-coding RNA species including long non-coding RNAs (lncRNAs), pseudogenes, and circular RNAs (circRNAs) [32,33,34,35,36]. This ability of microRNAs to target thousands of coding and non-coding RNAs suggests that they may function as key nodes in complex, inter-connected post-transcriptional RNA/RNA networks.

## 3. Webs of Co-Regulatory miRNA/Target Interactions May Cause Ripple Effects on Transcriptomic Equilibrium

**Figure 1:**
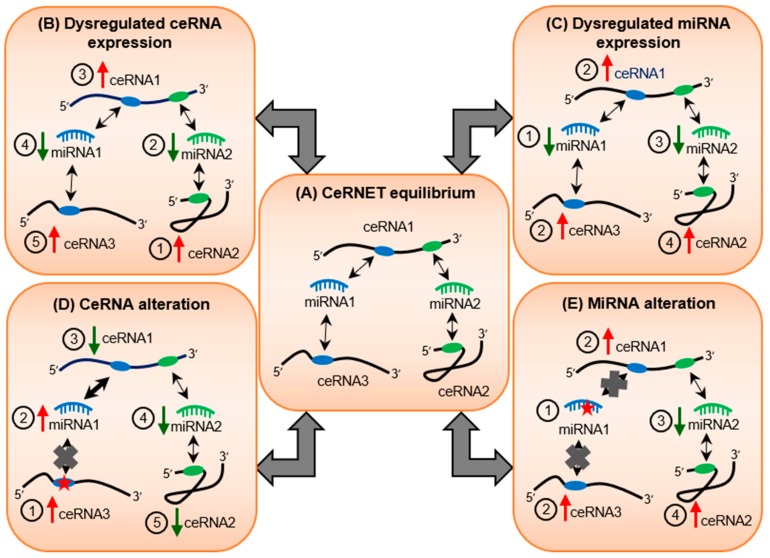
Potential impact of RNA and miRNA alterations on competing endogenous RNA (ceRNA) network (ceRNET) equilibrium. The ceRNET equilibrium is maintained by optimal molecules of ceRNAs and miRNAs (**A**). Changes in the expression of ceRNA1 (**B**) or miRNA1 (**C**) shift the ceRNET balance, leading to the dysregulation of ceRNA levels and miRNA activity, as indicated by red arrows (indicate upregulation) and green arrows (indicate downregulation). Nucleotide alterations (indicated by red stars) on ceRNA3 (**D**) or miRNA1 (**E**) could transform the miRNA-targeting landscape and ceRNET integrity. Double-headed arrows indicate bi-directional interaction; grey crosses indicate loss of interaction; numbers in the circles indicate the sequence of events.

Over the last two decades, a vast body of literature has characterized how specific miRNAs regulate key target transcripts in physiological and disease settings. However, significantly less attention was focused on the potential effect that miRNA targets had on regulating miRNA activity. In recent years, multiple independent groups have described the existence of an additional layer of miRNA-dependent regulation: transcripts that contain binding sites for common miRNAs can modulate the available pool of miRNAs and thus reciprocally regulate each other, thus acting as miRNA ‘sponges’ or competing endogenous RNAs (ceRNAs) [37,38,39,40,41,42]. For example, an increase in the expression of a specific RNA would sponge miRNAs away from its ceRNAs, subsequently leading to their upregulation [43]. This interaction is further complicated as each RNA is often targeted by multiple miRNAs, creating a ceRNA network (ceRNET) that bridges numerous independently expressed RNAs and miRNAs into an intricately connected web (Figure 1, middle panel). In physiological conditions, the stoichiometric balance between RNA and miRNA molecules is crucial in maintaining transcriptomic homeostasis [44]. Any alteration in ceRNA or miRNA expression could lead to the subsequent perturbation of ceRNETs (Figure 1, top panels). In addition, RNA alterations that affect miRNA target recognition could abolish and/or create miRNA binding sites, leading to the disruption of ceRNET equilibrium (Figure 1, bottom panels).

The exquisite sensitivity of ceRNA networks coupled with their potential butterfly effect on transcriptomic equilibrium has led to spirited debate about the optimal conditions for ceRNA crosstalk. In particular, the affinity for specific target sites, endogenous miRNA/target pool ratios, and cooperative binding of proximal sites for the same or different miRNAs have been shown to be key determinants for optimal ceRNA crosstalk [45,46,47,48]. Although much remains to be discovered about the stoichiometry, subcellular localization, and other factors underpinning the effectiveness of ceRNA regulation, a tremendous body of literature from multiple studies has identified specific miRNA/ceRNA networks that are key players in the development of several cancers. These have been extensively reviewed elsewhere [49,50,51,52].

**Figure 2:**
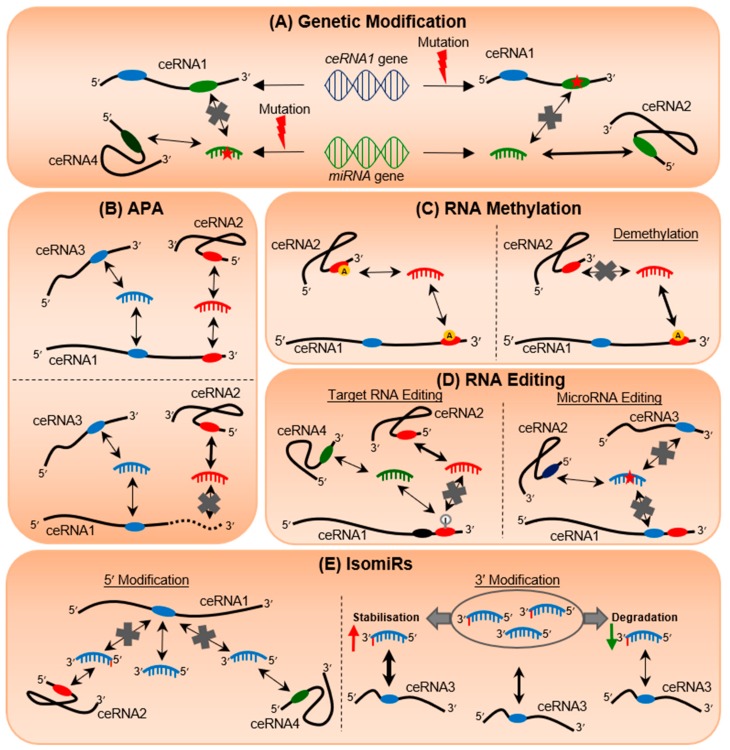
The butterfly effect of RNA alterations on transcriptomic equilibrium. The different modifications affecting either target RNAs or miRNAs and/or both, such as genetic modification (**A**), alternative polyadenylation (APA) (**B**), RNA methylation (**C**), RNA editing (**D**), and isomiR production (**E**). Ovals represent the miRNA response elements (MREs); double-headed arrows represent bi-directional regulation in which the thickness indicates the strength of the regulation; single-headed arrows indicate increase (pointed up) or decrease (pointed down) in RNA expression; red stars indicate non-specific nucleotide alteration; grey crosses indicate loss of interaction; red lightning bolts represent mutation events; yellow circles with an A represent methylation marks; grey circle with an I represents A-to-I RNA editing.

In recent years, significant breakthroughs have advanced our understanding into multiple aspects of RNA regulation and processing, which have profound implications for RNA stability, sequence composition, potential susceptibility to ceRNA crosstalk, and subsequent clinical applications. In this review, we will summarize the different alterations that may affect miRNA or target transcript expression and function, and thus have a wider effect on their associated ceRNETs. These include genetic modification, alternative polyadenylation (APA), RNA methylation, RNA editing, and isomiR production (Figure 2).

## 4. Genetic Mutations Can Alter Post-Transcriptional Regulation

Genomic instability is one of the key hallmarks of cancer [53]. Genetic mutations could occur in miRNA loci or that of their target transcripts. In addition to disrupting protein structure and function, mutations may significantly disrupt miRNA/target interactions. It has been shown that a point mutation within the *MIR128B* gene, RS4;11, reduces miR-128b processing, its mature miRNA expression, and subsequently results in glucocorticoid resistance in acute lymphocytic leukemia [54]. This study underscores the importance of structural requirements for pri-miRNA processing and the role of miRNA biogenesis in carcinogenesis.

Point mutations within miRNA genes are not random, isolated events [55]. Systematic analysis by Gong et al. [56] demonstrated that the majority of single nucleotide polymorphisms (SNPs) at the stem region of precursor miRNAs (pre-miRNAs) reduce mature miRNA expression by reducing the stability of hairpin structures. On the other hand, an A/G polymorphism (rs895819) within *MIR27A* increases the mature miR-27a levels and reduces the expression of its target *ZBTB10*, leading to an increased risk of gastric cancer [57]. It has also been found that a few SNPs occur at miRNA seed regions, which are key determinants for target recognition, thus potentially altering the spectrum of targets for that particular miRNA [56]. Moreover, as the seed is not the only determinant for target affinity, SNPs at non-seed regions of miRNAs may also affect miRNA-target interactions as well as miRNA stability.

In addition to SNPs on miRNAs, SNPs within miRNA target transcripts may modulate the landscape of miRNA/target interactions. Global analysis of SNPs in large population cohorts identified approximately 250 SNPs that occur at evolutionarily conserved miRNA target sites, which may alter or abolish miRNA binding [55]. For example, a SNP (rs1434536) on the 3′UTR of bone morphogenic receptor type 1B (BMPR1B) creates C and T alleles that are differentially targeted by miR-125b in breast cancer [58]. SNPs may also affect cancer susceptibility: an SNP on the 3′UTR of the critical proto-oncogene *KRAS* leads to the loss of let-7-mediated repression and subsequent upregulation of KRAS in non-small cell lung cancer (NSCLC) patients [59].

Point mutations in miRNA target genes can be harnessed to develop powerful, targeted cancer therapies. Acunzo et al. [60] devised miRNA-like artificial molecules (amiRNAs) and showed that amiR-KS3 specifically targets mutant *KRAS* transcripts carrying the G12S mutation without affecting wild-type transcripts in NSCLC. This amiR-KS3 treatment was also found to be effective in other cancers associated with G12S mutation. As more than 30% of all human cancers, including 95% of pancreatic cancers and 45% of colorectal cancers, are driven by *RAS* family mutations, these amiRNAs specific to oncogenic *KRAS* may represent a significant breakthrough towards using miRNA drugs as personalized therapies for cancer.

## 5. 3′UTR Shortening Transforms the miRNA Regulatory Landscape

In addition to genetic mutations, alterations in post-transcriptional regulatory processes can also transform miRNA/target interactomes. The majority of identified miRNA binding sites are in the 3′UTRs of target transcripts, which were once thought to be unimportant as they did not possess protein-coding potential. Now established as critical regulatory regions of mRNAs, 3′UTRs frequently undergo alternative cleavage and polyadenylation (APA), which results in the loss or gain of multiple miRNA binding sites. Genome-wide studies have revealed a high prevalence of APA: 50–70% of human mRNAs possess multiple 3′UTR isoforms [61,62].

APA-mediated 3′UTR shortening has been described in multiple cancers [63,64,65,66,67,68,69,70,71,72,73,74,75]. NUDT21, a key regulator of APA, is frequently downregulated in several cancers [66,67,69,70,76]. Knockdown of NUDT21 causes 3′UTR shortening of various oncogenes by increasing the usage of proximal polyadenylation sites, leading to significant increases in cell proliferation, migration, and xenograft growth [66,68,70]. Conversely, restoration of NUDT21 expression protected the proximal actual polyadenylation site (PAS) from cleavage by CPSF, leaving only the cleavage of distal PAS. This increased miRNA-mediated transcript repression, and thus decreased cancer growth.

Oncogene transcripts that undergo 3′UTR truncation often exhibit enhanced oncogenic properties [77,78]. Mechanistically, the shorter 3′UTR isoforms exhibit greater stability, and subsequently higher protein expression than the corresponding full-length transcripts, in part because of the loss of miRNA binding sites [63,65,79,80]. These studies in cell lines were corroborated by a study by Andres and colleagues [65], who found that 3′UTR shortening of insulin-like growth factor mRNA binding protein 1 (IGF2BP1) led to the loss of let-7 regulation, resulting in elevated IGF2BP1 expression and accelerated liver metastasis in colorectal cancer patients. Multiple studies have demonstrated that the loss of miRNA regulation on the shortened 3′UTRs of oncogenes promotes tumor phenotypes in several cancers, thus suggesting that it is a widespread mechanism by which oncogenes evade post-transcriptional repression [79,81,82,83,84].

3′UTR shortening also has a ripple effect on the associated ceRNA networks, as the loss of MREs increases the available pool of miRNAs, shifting miRNA-directed repression onto the wider ceRNA network and decreasing ceRNA expression (Figure 2B). This has been observed in breast cancer cell lines, where the shortening of the *PTEN* 3′UTR causes the downregulation of its ceRNA *EPS15*, while *PHF6* 3′UTR shortening decreases the expression of its ceRNA *YOD-1* [85].

In addition to the loss of RNA/RNA interactions, 3′UTR shortening also results in a loss of RNA/protein interactions with RNA binding proteins (RBPs) [86]. For example, the long 3′UTR of *CD47* is associated with HuR and SET, facilitating the translocation of CD47 protein to the plasma membrane. In contrast, the short 3′UTR isoform has fewer HuR binding sites, resulting in CD47 protein localization to the endoplasmic reticulum [86]. As RBPs regulate transcripts at multiple stages of their life cycle, it is likely that the loss of RBP regulation on shortened 3′UTR transcripts could have a variety of effects on transcript expression and downstream protein function. Clinically, APA has shown promise as a prognostic marker, whereby the different clusters of APA have been used successfully to predict survival outcome of lymphoma patients [73] and relapse in prostate cancer patients [87].

## 6. RNA Methylation Regulates Epitranscriptomic Plasticity

In the 1970s, the discovery of modifications on specific nucleotides on the mRNAs of rat Novikoff hepatoma cells, mouse myeloma, mouse L cells, hamster cells, and HeLa cells sparked tremendous interest in the potential plasticity of RNA [88,89,90,91,92]. Since then, RNA methylation on the adenosine base at the nitrogen-6 position (also known as m6A) has been shown to be the most abundant methylation event on eukaryotic mRNAs [88,92]. In contrast to permanent alterations such as genetic mutation and APA, which cause irreversible changes to RNA sequence and structure, the m6A modification represents a reversible mechanism that provides functional diversity by modulating RNA structure, RNA/protein interactions, and cellular localisation [93,94].

Similar to epigenetic regulation, epitranscriptomic regulators consist of writers, readers, and erasers, which encode, decode, and remove the RNA methylation mark, respectively [93]. Researchers have started to unravel the impact that the dysregulation of RNA methylation plays in cancers. Major m6A methyltransferases (writers) include methyltransferase-like 3 (METLL3) and methyltransferase-like 14 (METLL14). High METTL3 expression has been associated with poor prognosis of hepatocellular carcinoma (HCC) patients [95]. The increased expression of METTL3 increases m6A abundance, and its growth promoting effect has been validated in a few cancer types [96,97,98]. METTL3 expression is increased in most cancers, while the dysregulation of METTL14 expression is cancer-dependent [99]. Another well-studied regulator of RNA methylation is fat mass and obesity-associated protein (FTO), which removes the m6A mark from mRNAs [100]. Increased expression of FTO enhances tumorigenesis of breast cancer as well as acute myeloid leukemia (AML), and is associated with poor prognosis of gastric cancer patients [101,102,103]. Recent efforts have focused on the development of therapies targeting the m6A reader YTHDF2, which selectively inhibits the growth of leukemic stem cells without affecting the proliferation of hematopoietic stem cells in a mouse model of AML [104].

The m6A mark is frequently enriched in polyadenylated (poly(A)) mRNAs, particularly near the stop codon. This suggests the possible involvement of m6A in modulating translation termination [92,105,106,107]. In addition, m6A marks are enriched in 3′UTRs, suggesting that they may influence miRNA-mediated regulation [106,108]. Interestingly, miRNAs may regulate de novo m6A methylation. Specifically, mutations within the miRNA seed region abolish m6A marks on the original targets and increase m6A peaks on the mRNAs targeted by the mutated miRNA [108]. Further studies have unravelled the involvement of m6A in controlling RNA stability and translation [98,109,110]. Knockdown of Mettl3 or Mettl14 decreases the methylation status, but increases *Igf3bp3* transcript stability in mouse embryonic stem cells, owing to the association of HuR with the demethylated sites, which blocks miRNA binding [109]. 

In addition to mRNAs, m6A marks have been detected on miRNAs and significantly influence miRNA expression and function. The m6A marks on pri-miRNAs are read by HNRNPA2B1, which interacts with DGCR8 and promotes pri-miRNA processing in the nucleus [111]. Furthermore, the steady-state levels of several miRNAs are dysregulated upon the downregulation of the m6A demethylase FTO [112]. Most recently, Konno et al. [113] discovered that the high expression of METTL3 and METTL14 is associated with increased methylation of mature miR-17-5p in pancreatic cancer patients compared with paired normal tissues. They also observed that the methylation level of miR-17-5p in serum samples could identify early stage pancreatic cancer patients, hence making it a potential diagnostic tool [113]. Although it is now clear that methylation can modulate miRNA expression, the impact of this on miRNA targeting ability has yet to be examined. It would be intriguing to explore this area and its effect on transcriptomic equilibrium as well as cancer development.

Other classes of ncRNAs have also been shown to be regulated by methylation. Warda et al. [114] identified METTL16 as a m6A methyltransferase, which methylates U6 small nuclear RNA (snRNA) and potentially other lncRNAs. A few studies have started to uncover the importance of lncRNA methylation. For example, methylation of a subset of human box C/D snoRNAs hinders the binding of the 15.5 kDa protein and affects RNA folding [115]. On the other hand, the m6A mark on the lncRNA MALAT1 induces a conformation change that facilitates binding of the RBP HNRNPC [116]. In pancreatic cancer, demethylation of the lncRNA KCNK15-AS1 by ALKBH5, a RNA demethylase, inhibits migration and invasion [117]. In summary, methylation has diverse, widespread effects on both coding and non-coding RNAs, which contributes to cancer development when it is disrupted. In addition, regulators of RNA methylation have great potential as diagnostic and prognostic tools for cancer. Further investigation into the therapeutic potential of these regulators could provide novel avenues for cancer treatment.

## 7. RNA Editing Rewires RNA Communication Networks

RNA editing is an additional type of RNA processing that contributes to transcriptome diversity. RNA editing of target transcripts generates a pool of heterogenous edited and wild-type RNAs. This may result in the synthesis of mutant proteins, which vary in structure and function, or the alteration of miRNA/target interactomes [118,119,120,121]. Large-scale RNA-sequencing data coupled with ultradeep sequencing of selected Alu sequences has revealed millions of RNA editing sites in the human transcriptome, suggesting that RNA editing is a widespread phenomenon [122,123]. Aberrant changes in RNA editing disrupt the equilibrium of edited and non-edited RNAs, leading to transcriptomic drift and subsequently altering protein products and/or expression in many cancers [121,123,124,125,126].

The most frequent and well-characterized RNA editing event is adenosine (A) to inosine (I) conversion, which is mediated by the adenosine deaminase acting on RNA (ADAR) enzyme family [127]. In this family, ADAR1 is the most abundant and ubiquitous member, which has been shown to be upregulated in multiple cancers [128,129]. Another type of RNA editing is cytidine (C) to uridine (U) editing, which is catalysed by the catalytic deaminase, apolipoprotein B mRNA editing enzyme, catalytic polypeptide 1 (APOBEC1) [130].

The dysregulation of RNA editing in cancers prompted the study of its clinical relevance. One of the most frequently edited genes in cancer is antienzyme inhibitor 1 (*AZIN1*). High levels of *AZIN1* RNA editing have been observed in several cancer types [120,131,132,133,134,135]. In addition, *AZIN1* RNA editing has been shown to be a potential prognostic biomarker for overall survival and an independent risk factor for lymph node and distant metastasis in some cancers [131,132,133]. Moreover, Han et al. [126] found a correlation between RNA editing with drug sensitivity, in which the sensitivities of paclitaxel, irinotecan, and topotecan are associated with editing in *AZIN1*.

In addition to protein re-coding events, RNA editing may enable 3′UTRs to escape miRNA-mediated regulation or create novel miRNA binding sites [136,137]. For example, an extensive A-to-I RNA editing mediated by ADAR1 on the *ARHGAP26* 3′UTR increases both RNA and protein expression of ARHGAP26 by abolishing the repressive effects of miR-30b-2p and miR-573 in multiple human cancer cell lines [138]. A study by Zhang et al. [139] found that the effect of RNA editing on miRNA-mediated repression is affected by the number of editing sites, and this may be cancer-specific. As miRNAs also target the coding regions of target transcripts, editing events in these regions could also result in changes in miRNA regulation in addition to the alterations in protein structure and function. Any changes in miRNA binding on the edited sites would affect miRNA bioavailability, which in turn will cause ripple effects in associated ceRNETs. Interestingly, RNA editing on miRNA targets tends to be more prevalent in tumor cells. This suggests that RNA editing could be a gain-of-function mutation, which promotes tumorigenesis [123].

MiRNAs themselves are heavily edited by ADAR enzymes in cancer. Multiple miRNA editing sites have been discovered, and half of them are widespread in several tissues [140]. Similar to the effect of SNPs on miRNA biogenesis, post-transcriptional editing of primary miR-376a-1 as well as mature miRNAs affect miRNA hairpin stability [141], while RNA editing of miR-222/221 and precursor miR-21 disrupts miRNA maturation and expression in glioblastoma [142]. More importantly, miRNA editing could redirect miRNA target recognition [143]. For example, miR-200b is a tumor suppressive miRNA that targets *ZEB1*/*ZEB2*, while edited miR-200b is an oncomiR that not only loses its wild-type function, but also gains a new role in targeting *LIFR*, thus promoting cell migration and invasion of multiple cancer cell lines [144]. Recent profiling of miRNA editing levels using 10,593 miRNA-seq samples from The Cancer Genome Atlas (TCGA) detected a global reduction in miRNA editing events in cancers. Consistently, head and neck squamous cell carcinoma patients with higher miR-376a-2 editing levels exhibit better prognosis than those with low editing levels [123].

In addition to editing of mRNAs and miRNAs, global analysis of A-to-I RNA editing events in 17 cancer types from TCGA detected widespread RNA editing on lncRNAs across different cancers [126,145]. Recent development of LNCediting, a lncRNA secondary structure prediction tool, could predict the impact of A-to-I RNA editing on lncRNA secondary structures, as well as the potential alterations in miRNA–lncRNA interactions [145]. The functional implications of RNA editing on lncRNAs have yet to be discovered, but with our current knowledge of the importance of lncRNAs in cancer, we postulate that RNA editing of lncRNA may contribute to tumorigenesis. In the future, it may also be worthwhile to study the effects of other RNA editing types, such as C-to-U and their contribution to post-transcriptional regulation and potential impact on cancer.

## 8. IsomiRs Add Tremendous Diversity to miRNA Species and Targetome Regulation

It was originally thought that each miRNA precursor would produce a maximum of two mature miRNAs, one from each arm of the hairpin. However, we now know that substitution, insertion, or deletion of nucleotide(s) at the 5′- or 3′-end of miRNAs can generate different isoforms, or isomiRs. A few studies have shown that isomiRs may be derived from differential DICER or DROSHA cleavage within the precursor miRNA [146] or DICER-independent cleavage by AGO2, giving rise to polymorphic isomiRs as well as templated or nontemplated 5′ isomiRs and 3′ isomiRs [147,148].

Intriguingly, isomiRs exhibit highly specific expression in normal and cancer samples, highlighting their potential as tumor biomarkers [149,150,151,152]. IsomiR expression may be dependent on gender, race, and population [149,153,154]. Recently, it has been demonstrated that the presence of 5′ isomiRs could effectively classify 32 different tumor types with more than 90% sensitivity [150,155]. Findings from Telonis et al. [149] and Lan et al. [156] support the advantages of using isomiRs in the classification of breast cancer subtypes compared with protein-coding gene expression. High levels of isomiRs hsa-let-7i-5p | 3′a-1 and hsa-miR-197-3p | 3′a-1 have been found in ER negative tumors, but not in ER positive tumors [156]. On the other hand, the 5′ isomiR hsa-miR-93-5p | 5′t-1 disrupts the estrogen signalling pathway by targeting SHC4, while hsa-miR-27a-3p | 5′t-1, hsa-miR-92a-1-3p | 5^′^-1, and hsa-miR-106b-3p | 5′t-1 interfere with mitogen-activated protein kinase (MAPK) signalling by targeting MAPK14, MAPK8, and RAP1B, respectively, in the ER positive tumors, showing the high specificity of these isomiRs in cancer subtype classification [156].

IsomiRs have also been shown to be important in other cancer types. Some isomiRs such as miR-497 and miR-21 display a diverse distribution pattern in gastric cancer compared with normal gastric tissue [151]. Analyses of RNA sequencing and small-RNA sequencing data from 10 cancer types available in TCGA have found a significantly lower miR-21 degradation ratio and adenylation ratio in the tumor samples that are mediated by PAPD5 [152]. A study on 74 prostate cancer patients has detected high expression of 3′ isomiRs of miR-21, miR-204, and miR-374 in extracellular vesicles in urine [157]. Compared with the mature form of these miRNAs, 3′ isomiR expression analysis exhibited higher sensitivity (72.9% versus 70.8%), higher specificity (88% versus 72%), and area under the curve (0.866 versus 0.766), hence suggesting that they may be robust, non-invasive biomarkers for prostate cancer.

Both 5′ and 3′ modified isomiRs could contribute to transcriptomic disequilibrium in different ways (Figure 2E). As the seed region at positions 2 to 8 from the 5′-end of miRNAs plays a key role in target recognition, the insertion and/or deletion of nucleotides at the 5′-termini alters seed regions and targeting spectrum, resulting in 5′ isomiRs that recognize novel targets [158]. This may cause a transformation in the miRNA targeting landscape as a 5′ isomiR could dilute the targeting effect of unmodified miRNA on ceRNA1, while at the same time disrupting the stability of other ceRNETs by regulating different ceRNAs (ceRNA2 and ceRNA4) (Figure 2E, left panel). For example, the 5′ isomiR-140-3p has higher expression than its canonical miR-140-3p and has gained the ability to target *COL4A1, ITGA6,* and *MARCKSL1* [159].

On the other hand, it has been shown that 3′ modifications modulate miRNA stability [156,160]. For example, 3′ adenylation of miR-21 by PAPD5 (also known as TUT3) accelerates miR-21 degradation [152], while 3′ adenylation of miR-122 by PAPD4 (also known as TUT2) stabilizes the mature form of miR-122 in the liver [160]. Furthermore, 3′ uridylation by ZCCHC11 (also known as TUT4) destabilizes miR-26 expression, which subsequently reduces miR-26’s repressive effect on IL-6 [161]. As depicted in Figure 2E (right panel), the increase or decrease in miRNA levels upon 3′ modification may affect the effectiveness of their target regulation. This could lead to differential target expression and modulation of related RNA/RNA networks. Even though the 3′ modification does not affect the seed region, Yang and colleagues [162] found that 3′ uridylated miR-27 is able to associate with RISC and repress non-canonical targets in HEK293T and HeLa cells.

The discovery of isomiRs has led to a paradigm shift away from the canonical “one arm-one miRNA” hypothesis, and revealed our limited understanding about miRNA biogenesis and the repertoire of effects caused by its modification [149]. Critically, it also underscores the importance of mining the wealth of information in RNA sequencing datasets beyond the standard pipelines analysing annotated transcripts [146]. The presence of isomiRs also diversifies the cellular transcriptomic targetome as isomiRs from the same hairpin arm have differential target recognition ability [149]. Cancer cells may harness this mechanism to preferentially upregulate and stabilize oncomiR expression, thus enhancing tumorigenic potential. With growing evidence about the importance of isomiRs, it is crucial to identify different isomiRs and understand their regulation and roles in normal and disease conditions.

## 9. RNA-Binding Proteins (RBPs), the Silent Guardians of the Transcriptome

In addition to RNA/RNA interactions, almost all RNA species are extensively regulated by RNA/protein interactions. RNA-binding proteins (RBPs) control multiple stages of the transcript life cycle, from transcription to processing, localization, translation, and degradation, and have been shown to act as key regulators of both coding and non-coding RNAs in diverse cellular processes [163]. Some RBPs may synergistically or competitively interact with each other to control gene expression. For example, the mediation of association between eIF4E and the mRNA cap by polyadenylation binding protein (PABP) leads to the stabilization of newly transcribed mRNA and the recruitment of ribosomes to initiate translation [164,165]. On the other hand, a competitive interaction between CELF1 and HuR at the *MYC* transcript 3′UTR regulates myelocytomatosis (MYC) translation in the intestinal mucosa [166]. 

Owing to their wide-ranging functions, RBPs have been shown to play crucial roles in different stages of cancer progression from proliferation to evasion of apoptosis, induction of angiogenesis, activation of epithelial to mesenchymal transition (EMT), and avoidance of immune surveillance [167]. Recently, a superfamily of RBPs known as the La related protein (LARP) family has gained a lot of attention in cancer research. This superfamily shares a well-conserved La motif (LAM) and is highly conserved throughout eukaryotic evolution [168]. Some members of this superfamily, such as LARP1 [169,170,171,172] and LARP6 [173], control the expression of target transcripts and promote the progression of several cancer types. On the other hand, LARP4A [174] and LARP4B [175] are tumor-suppressive RBPs, which inhibit cancer metastasis and growth, respectively. 

Many published studies have characterized the interaction of RBPs with one or several target transcripts. However, these may only capture a small subset of the RBP/targetome as transcriptome-wide mapping of RBP–RNA binding sites have shown that a single RBP may regulate hundreds or thousands of target transcripts [176,177,178]. For example, activation of the RBP negative elongation factor complex member E (NELFE) accelerates HCC progression by modulating the expression of transcripts that are transcriptionally induced by MYC [179]. 

To date, more than 1500 RBPs have been identified, and this number is expected to increase with the advancement of RNA interactome capture and immunoprecipitation techniques [180,181,182]. However, some questions remain to be addressed on the specific characteristics that define an RBP, as well as the mechanism, sequence features, and structural importance of the protein–RNA associations [182]. Potential competing endogenous crosstalk between RNAs that share binding sites for specific RBPs adds a further dimension to this aspect of post-transcriptional regulation. Analogous to the concept of miRNA/ceRNA interactions, RNAs may indirectly communicate with other RNAs through shared RBPs. At the same time, each RNA may be regulated by multiple RBPs, which may regulate it cooperatively or competitively at different stages of its life cycle. Therefore, any changes in RBP expression or binding affinity may result in a ripple effect that contributes to transcriptomic disequilibrium.

## 10. Crosstalk between RNA/RNA and RNA/Protein Interactions in Regulating Transcriptomic Equilibrium

**Figure 3:**
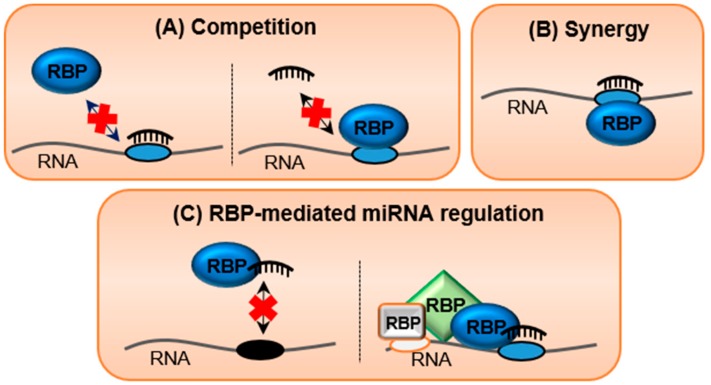
The multi-faceted crosstalk between RNA/RNA and RNA/protein interactions in post-transcriptional regulation. (**A**) Competitive interactions whereby miRNAs and RNA binding proteins (RBPs) compete for binding to a specific target region. (**B**) Synergistic interactions whereby miRNAs and RBPs bind cooperatively to a shared target region. The association of either miRNAs or RBPs facilitates the binding of the other. (**C**) RBPs may inhibit miRNA function by sequestering miRNAs away from the target site (left hand panel) or promote miRNA-mediated regulation by recruiting other RBPs onto the RNA. Ovals represent the miRNA and RBP binding sites; double-headed arrows represent bi-directional interaction between the binding site and RBPs or miRNAs; red crosses represent loss of interaction.

Post-transcriptional regulation of gene expression is analogous to traffic along a busy road. Many vehicles, such as ribosomes and RBPs, are constantly travelling or parked along the length of RNA transcripts and their activity can be regulated by policemen, the miRNAs. Occasionally, there may be reciprocal interactions, competitive or synergistic, between miRNAs and the RBPs in modulating the expression of their shared targets [183] (Figure 3). Many studies have validated the antagonistic roles of HuR and miRNAs in binding to the 3′UTRs of several target RNAs in cancers [82,184,185,186]. This leads to the increased expression of target transcripts and acceleration of oncogenic phenotypes (Figure 3A). Other RBPs such as coding region determinant-binding protein (CRD-BP) and DND microRNA-mediated repression inhibitor 1 (Dnd1) were also shown to antagonise miRNA binding on the shared mRNA targets [187,188]. On the other hand, HuR can synergistically interact with miRNAs. For example, the presence of HuR increases the binding affinity of let-7 to the *MYC* 3′UTR, thus reducing MYC expression [189] (Figure 3B). Binding of another RBP, Pumilo homolog 1 (PUM1), changes the structure of target RNAs and facilitates miRNA binding onto their MREs [190,191].

Interaction between miRNAs and multiple RBPs in the RNA-induced silencing complex (RISC) is required for miRNA-mediated repression (Figure 3C). For example, the miRNA-mediated interaction between AGO2, the key catalytic component of the RISC, and EDC4 causes mRNA decapping and degradation [192]. SILAC (Stable isotope labeling by amino acids in cell culture)-based mass spectrometry analysis revealed that AGO2 could interact with many RBPs, such as HuR, YBX1, HNRNPC, PABP1, PABP4, and LARP1, in a miRNA-dependent manner, as well as with DHX30, DHX36, IGF2BP1-3, and HNRNPL in a miRNA-independent manner [192]. In contrast, some RBPs may associate directly with miRNAs to interfere with their function (Figure 3C). For example, HuR binds to miR-21 upon inflammatory stimulus, leading to the sequestration of miR-21 from the 3′UTR of protein programmed cell death 4 (*PDCD4*) and the subsequent activation of apoptosis [193].

The intricate mesh of post-transcriptional regulation, which is tightly coordinated by constant communication between miRNAs, RBPs, and target RNAs, is finely balanced and any perturbation may promote disease development. A study by Suzuki et al. [194] emphasizes the importance of this crosstalk, in which targeting by miRNAs and small-interfering RNAs (siRNAs) of sites that overlap with RBP binding sites interferes with endogenous RBP activity. This interaction was termed “crosstalk with endogenous RBPs (ceRBPs)”. The widespread seed-to-RBP crosstalk contributes to seed-induced off-target effects and perpetuates the RNA interference (RNAi)-mediated growth phenotype. This study also shows that post-transcriptional regulation is a multi-layered and highly coordinated process that requires further exploration. Clinically, a deeper understanding of the reciprocal communication between miRNAs, RBPs, and target RNAs may potentially be used to predict off-target effects of cancer drugs, as well as to identify key nodes for novel therapeutic interventions.

## 11. Conclusions

The past twenty years of RNA biology research have led to a paradigm shift in our understanding of the essential roles that these biomolecules play in physiological and pathophysiological conditions and the biochemical and molecular mechanisms underlying their activity. This will continue into the future as novel species of RNAs or aspects of their regulation and function are discovered. To date, researchers have made significant advances in our understanding of the biogenesis, targeting dynamics, and mechanism of action of miRNAs in post-transcriptional regulatory networks. Deeper insights into the different mechanisms by which miRNAs and their targets are regulated and modified in specific spatial, temporal, or disease contexts will be critical to appreciate their physiological importance and potential as diagnostic, prognostic, and therapeutic targets for cancer management.

In this review, we discuss the different genomic, epitranscriptomic, and post-transcriptomic alterations that regulate both coding and non-coding RNAs, as well as their effects in tumorigenesis. The effect of these modifications may be more extensive than previously thought as the majority of the transcriptome may form webs of inter-connected competing endogenous RNA/RNA and RNA/protein interactions. Any perturbation of key miRNAs, RBPs, or target transcripts could significantly shift the steady state equilibrium, leading to subsequent perturbation of other players in the same network. Further research will be of critical importance to unravel the interplay between these complementary facets of post-transcriptional regulation and understand how their dysregulation may drive carcinogenesis.

Numerous studies have presented compelling results highlighting the key role of ncRNAs in driving cancer development, and multiple clinical trials are underway to evaluate their effectiveness as potential cancer therapeutics [195,196,197]. In 2018, the first siRNA drug, Patisiran, gained FDA (Food and Drug Administration) approval for clinical use. As such, although we still have much to learn about how various aspects of RNA processing regulate ncRNA function, we anticipate that a better understanding of the combinatorial effect of these alterations on specific ncRNAs may lead to breakthrough insights into ncRNA biology and pave the way for novel targeted therapies against cancer.
